# Prevalence and clinical characteristics of methicillin-resistant *Staphylococcus aureus* infections among dermatology inpatients: A 7-year retrospective study at a tertiary care center in southwest China

**DOI:** 10.3389/fpubh.2023.1124930

**Published:** 2023-03-14

**Authors:** Zhijian Yao, Yidan Wu, Hongming Xu, Ying Lei, Wanyu Long, Meixian Li, Yue Gu, Zhiwen Jiang, Cunwei Cao

**Affiliations:** ^1^Department of Dermatology and Venereology, The First Affiliated Hospital of Guangxi Medical University, Nanning, Guangxi, China; ^2^Guangxi Key Laboratory of Mycosis Prevention and Treatment, Nanning, Guangxi, China; ^3^Department of Clinical Medicine, Guangxi Medical University, Nanning, Guangxi, China

**Keywords:** methicillin-resistant *Staphylococcus aureus*, prevalence, comorbidity, antibiotic susceptibility, retrospective study

## Abstract

**Background:**

Increased rates of methicillin-resistant *Staphylococcus aureus* (MRSA) antibiotic resistance and the associated morbidity have increased dermatologists' attention to skin and soft tissue MRSA infections. However, the clinical characterization of MRSA skin and soft tissue infections (SSTIs) in Southwest China is lacking, which precludes optimal prevention and treatment of these infections.

**Objectives:**

This study was conducted to characterize the prevalence, clinical comorbidities and antibiotic susceptibility of MRSA isolates from SSTIs, including community-associated (CA) and healthcare-associated (HA) isolates.

**Methods:**

In the Dermatology Inpatient Department of the First Affiliated Hospital of Guangxi Medical University, a retrospective study was conducted on data, including patient demographics and clinical information, from culture-confirmed *S. aureus* isolated from skin and soft tissue between January 1, 2015, and December 31, 2021. Isolate susceptibility to 13 antibiotics was determined using the Vitek 2 system.

**Results:**

From among 864 *S. aureus* strains, we identified 283 MRSA (32.75%) isolates comprising 203 CA-MRSA and 80 HA-MRSA isolates. The average rate of CA-MRSA isolation for MRSA SSTIs was 71.73%. The HA-MRSA isolation rate for MRSA SSTIs increased significantly. HA-MRSA-infected patients were older. The most common dermatological presentation of CA-MRSA infection was staphylococcal scalded skin syndrome, while the comorbidity severe drug eruption was significantly associated with HA-MRSA infection. One CA-MRSA strain was resistant to linezolid, and one HA-MRSA strain had an intermediate phenotype for vancomycin; both strains had low sensitivity to clindamycin and erythromycin (3.70%~19.40%). However, HA-MRSA isolates were more susceptible to trimethoprim/sulfamethoxazole.

**Conclusions:**

CA-MRSA is a predominant pathogen causing SSTIs, and HA-MRSA infection incidence is increasing gradually. Both strains showed increasing antibiotic resistance. Our data on MRSA susceptibility may guide dermatologist antibiotic treatment decisions. Dermatologists should consider these identified comorbidities of MRSA SSTIs when patients are admitted and initiate early prevention and treatment of MRSA.

## 1. Introduction

*Staphylococcus aureus* is a common gram-positive bacterium that colonizes human skin and mucous membranes. *S. aureus* can cause disease ranging from skin and soft tissue infection (SSTI) to severe systemic infection, and its rate of resistance is rising (1). SSTI is one of the most common infections in the community and healthcare settings (2). *S. aureus* is the main pathogen implicated in SSTIs, which can cause folliculitis, impetigo, cellulitis and cutaneous abscesses (3).

Methicillin-resistant *S. aureus* (MRSA) emerged in the 1960s and spread worldwide, becoming the main cause of multidrug-resistant bacterial infections in hospital and community environments. MRSA infection places a tremendous burden on human health and the medical system, leading to higher mortality, longer hospital stays and higher medical expenses than methicillin-sensitive *S. aureus* (MSSA) infection (4). Moreover, MRSA strains insensitive to vancomycin and linezolid, which are less commonly isolated but pose a greater threat, have been reported in some countries worldwide (5, 6). The rate of MRSA SSTIs increased at an alarming rate in the United States at the beginning of the 21st century (7). In addition, the severity of MRSA SSTIs has been reported to have increased worldwide. However, the prevalence of MRSA SSTIs varies among regions and countries. The MRSA SSTI prevalence is <1% in some European countries and more than 60% in some regions of South America, Asia and the United States (8–11).

At present, the method most commonly used to distinguish HA-MRSA and CA-MRSA is based on epidemiological features (12–15). One important reason for this is that in clinical practice, HA-MRSA and CA-MRSA can be quickly distinguished by this method, which can be applied to retrospective studies with a large sample size (14, 15). Cases meeting the CDC definition of HA-MRSA include cases with (1) MRSA infection identified 48 h after admission to a hospital; (2) a history of hospitalization, surgery, dialysis, or residence in a long-term care facility within 1 year of the MRSA culture date; (3) the presence of a permanent indwelling catheter or percutaneous medical device at the time of culture; or (4) a known positive culture for MRSA before the study period. All other cases are classified as CA-MRSA (12, 13). The first community outbreak of HA-MRSA was reported in the United States in the 1990s, and such outbreaks have subsequently been reported worldwide (16). CA-MRSA infections were originally restricted to communities and included mainly SSTIs, but the number of CA-MRSA infections is now increasing in community and hospital settings (17, 18). Therefore, genotyping has been suggested as part of the epidemiological evaluation of HA-MRSA and CA-MRSA (12). For example, CA-MRSA contains *SCC*mec types IV, V, VI, and IX and mainly carries the Panton-Valentine leukocidin (PVL) gene, while HA-MRSA carries *SCC*mec types I, II and III (19). Increasing studies have shown that *SCC*mec types IV, V, and VII were the most frequent in SSTI-related MRSA (20). Previous studies showed that the overlap between the identification of HA-MRSA and CA-MRSA results obtained using epidemiological typing and genotyping (*SCC*mec sequence) could be satisfying, up to 81 and 85% for HA-MRSA and CA-MRSA respectively (13, 21). In particular, the overlap rate was even higher in MRSA SSTIs, with that of CA-MRSA reaching up to 93% (20). In this regard, the use of epidemiological typing to classify MRSA SSTIs is supported by the literature, indicating its clinical significance.

MRSA infection can be divided into three stages. The first and second stages of MRSA infection occur mainly in the skin and soft tissues, and the third stage is systemic infection (22). Therefore, it is very important to actively monitor and treat MRSA SSTIs before they progress to systemic infection. There is a lack of research data on the epidemiology and clinical characteristics of MRSA SSTIs in Southwest China. Our primary objective was to classify CA-MRSA and HA-MRSA isolates from SSTIs based on the definition criteria and then identify the epidemiology, clinical comorbidities and antibiotic susceptibility profiles of the isolates. In clinical practice, dermatologists can also quickly classify CA-MRSA and HA-MRSA isolates from SSTIs according to the definition criteria to better prevent and control MRSA SSTIs and better guide the selection of clinical antibiotics. To this end, we conducted a retrospective study over 7 years.

## 2. Materials and methods

### 2.1. Surveillance and inclusion criteria

Before data collection, this study was approved by the institutional review board at the First Affiliated Hospital of Guangxi Medical University (No. 2022-E430-01). A retrospective study was then conducted on data regarding *S. aureus* isolates collected from skin culture annually between January 1, 2015, and December 31, 2021. Skin culture isolates were collected from patients seen at the Dermatology Inpatient Department of the First Affiliated Hospital of Guangxi Medical University (Nanning, China). For patients with more than one *S. aureus* isolate during the study period, to avoid repeated collection of data, we collected data for only the first *S. aureus* isolate for each patient. The data obtained from the microbiology laboratory database matched the data of the electronic medical record in the hospital information system (HIS), and at least two independent investigators conducted a systematic review and reexamination.

Demographic information and clinical comorbidities were retrieved from the HIS according to the International Classification of Diseases, Tenth Revision (ICD-10) codes. If the patient's medical record showed more than one comorbidity, we chose the more severe comorbidity that can impair skin barrier function. We defined HA-MRSA and CA-MRSA infections according to the proposal of CDC and Naimi et al. (12, 13). HA-MRSA cases were defined for patients with (1) a MRSA infection identified 48 hours after admission to a hospital; (2) a history of hospitalization, surgery, dialysis, or residence in a long-term care facility within 1 year of the MRSA culture date; (3) the presence of a permanent indwelling catheter or percutaneous medical device at the time of culture; or (41) a known positive culture for MRSA before the study period. All other cases were classified as CA-MRSA. Patients without a *S. aureus*-related primary diagnosis and complications during hospitalization were classified as having *S. aureus* colonization (23), and patients with incomplete medical records were excluded. The assessment of clinical outcome among patients with MRSA SSTIs was a secondary objective. The endpoint of the assessment was the day of discharge. Clinical outcome was based on a composite assessment of overall clinical, serological, skin barrier repair, and skin secretions bacterial culture data obtained from the HIS, and was evaluated based on the following scale: (1) “cure” (resolution of clinically significant signs and symptoms associated with admission infection), (2) improvement (partial resolution of clinical signs or symptoms of infection), (3) aggravation (Uncontrolled clinical signs and symptoms of infection) and (4) death. Clinical effectiveness was defined as clinical cure or improvement.

### 2.2. Bacterial identification

Clinical isolates of *S. aureus* from patients were classified as either MSSA or MRSA by cefoxitin screening and oxacillin minimum inhibitory concentration (MIC) determination by the Vitek 2 system (bioMérieux, Marcy-l'Étoile, France) (24). Isolates resistant oxacillin (MIC > 4 μg/mL) or cefoxitin (>32 μg/mL) were considered MRSA. We determined the susceptibility and MIC values of each isolate to 13 antibiotics (vancomycin, teicoplanin, tigecycline, linezolid, daptomycin, rifampin, trimethoprim-sulfamethoxazole, gentamicin, moxifloxacin, levofloxacin, clindamycin, erythromycin, and penicillin) using the Vitek 2 system. The routine laboratory results were reported as susceptible, intermediate, or resistant to the antimicrobial agents tested.

### 2.3. Statistical analysis

We analyzed nominal variables with the chi-square test and continuous variables with Student's *t*-test. To evaluate the trend in the annual antibiotic sensitivity data, the chi-square test was used to calculate the statistical significance of differences. For comparison, we grouped the data into two 3-year study periods: January 2015 to December 2017 (the prior 3 years) and January 2018 to December 2020 (the most recent 3 years). All analyses were 2-tailed, and *P* < 0.05 was considered to indicate statistical significance. Statistical analysis software (SPSS, version 22.0; SPSS Inc.) was used for all calculations.

## 3. Results

### 3.1. Prevalence and relative proportions of MRSA and MSSA

**Figure 1:**
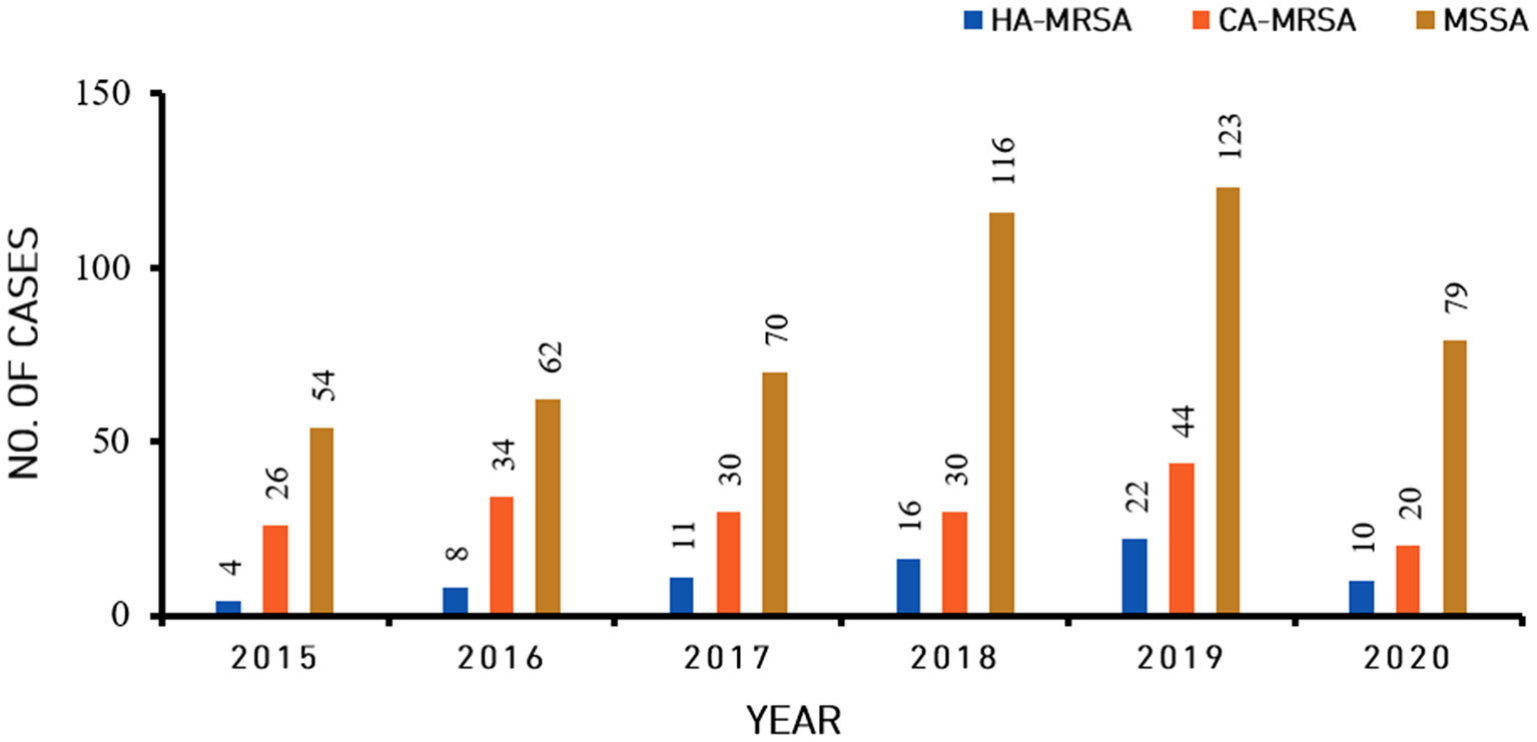
Incidence of community-associated methicillin-resistant *Staphylococcus aureus* (CA-MRSA), healthcare-associated methicillin-resistant *S. aureus* (HA-MRSA) and methicillin-sensitive *S. aureus* (MSSA) isolates from skin and soft tissue infections from 2015 to 2020.

In total, 864 *S. aureus* isolates were analyzed between January 1, 2015, and December 31, 2021. Among these isolates, the overall relative proportion of MRSA was 32.75% (283/864), and the overall relative proportion of MSSA was 67.25% (581/864). The 283 MRSA isolates consisted of 80 HA-MRSA isolates (80/283, 28.27%) and 203 CA-MRSA isolates (203/283, 71.73%) (Figure 1 and Table 1). During the last 1 year of the study, the relative proportion of MRSA was 26.67% (28/105), while the relative proportion of MSSA was 73.33% (77/105). The relative proportion of HA-MRSA among the MRSA isolates from January 1, 2018, through December 31, 2020, was significantly higher than the relative proportion from January 1, 2015, through December 31, 2017 (20.35% vs. 33.80%, *P* = 0.017).

**Table 1:** The number of patients with skin and soft tissue *Staphylococcus aureus* infection, including that by methicillin-resistant *S. aureus* (MRSA), healthcare-associated (HA) MRSA, and community-associated (CA) MRSA, by year.

**Year**	**HA-MRSA**	**CA-MRSA**	**MSSA**	**Total**
2015	4	26	54	84
2016	8	34	62	104
2017	11	30	70	111
2018	16	30	116	162
2019	22	44	123	189
2020	10	20	79	109
2021	9	19	77	105
Total	80	203	581	864

MSSA, methicillin-sensitive Staphylococcus aureus.

### 3.2. Demographic characteristic data

Table 2 summarizes the demographics and characteristics of HA-MRSA and CA-MRSA SSTIs. The median age of patients with HA-MRSA or CA-MRSA SSTIs was 49.80 years (range, 1 month to 81 years) or 38.97 years (range, 2 months to 89 years), respectively; those with HA-MRSA SSTIs were significantly older (*P* < 0.001). There was no significant difference in the distribution of HA-MRSA and CA-MRSA by sex.

**Table 2:** Comparison of the demographics of skin and soft tissue healthcare-associated methicillin-resistant *S. aureus* (HA-MRSA) and community-associated methicillin-resistant *S. aureus* (CA-MRSA) infections.

**Parameter**	**HA-MRSA (*n =* 80)**	**CA-MRSA (*n =* 203)**	***T/χ^2^***	***P-*value**	OR^*^	**95% CI**
Age (mean ± S, years)	49.80 ± 19.39	38.97 ± 25.91	3.4	<0.001		
<18 years (*n*, %)	8 (10)	58 (28.57)				
≥18 years (*n*, %)	72 (90)	145 (71.43)	2.4	0.1	0.6	0.343–1.138
Sex (F/M)	44/36	110/93	0	0.9	1	0.614–1.738

CI, confidence interval; OR, odds ratio.

* OR from the univariate analysis.

### 3.3. Comorbidities associated with HA-MRSA and CA-MRSA SSTIs

Table 3 lists comorbidities associated with HA-MRSA and CA-MRSA SSTIs. Comorbidities were associated with HA-MRSA, with the top 3 in descending order being pemphigus (26.25%), drug eruption (12.50%), and pustular psoriasis (11.25%). The rate of various comorbidities associated with HA-MRSA did not change significantly between the prior 3 years (2015.1.1–2017.12.31) and the most recenct 3 years (2018.1.1–2020.12.31).

**Table 3:** Comparison of comorbidities associated with skin and soft tissue healthcare-associated methicillin-resistant *S. aureus* (HA-MRSA) and community-associated methicillin-resistant *S. aureus* (CA-MRSA) infection from January 1, 2015 to December 31, 2021.

	**HA-MRSA**	**CA-MRSA**			
	**(*n =* 80)**	**(*n =* 203)**			
**Diagnosis**	**No. (%)**	**No. (%)**	***P-*value^※^**	**OR[Table-fn TN8]**	**95%CI**
Pemphigus^*^	21 (26.25%)	54 (26.60%)	0.952	0.982	0.546–1.767
Staphylococcal scalded skin syndrome	2 (2.50%)	32 (15.76%)	0.002	0.137	0.032–0.586
Drug eruption	10 (12.50%)	13 (6.40%)	0.091	2.088	0.876–4.977
Severe drug eruption^#^	9 (11.25%)	5 (2.46%)	0.006	5.02	1.627–15.482
Non-severe drug eruption^§^	1 (1.25%)	8 (3.94%)	0.432	0.309	0.038–2.208
Pustular psoriasis	5 (6.25%)	12 (5.91%)	1	1.061	0.361–3.115
Erythroderma	3 (3.75%)	10 (4.92%)	0.912	0.752	0.201–2.806
Dermatomyositis	4 (5.00%)	5 (2.46%)	0.472	2.084	0.545–7.969
Systemic lupus erythematosus	3 (3.75%)	5 (2.46%)	0.849	1.543	0.360–6.613
Eczema	0 (0.00%)	8 (3.94%)	0.161	-	-
Atopic dermatitis	3 (3.75%)	6 (2.96%)	1	1.279	0.312–5.243
Pyoderma gangrenosum	3 (3.75%)	6 (2.96%)	1	1.279	0.312–5.243
Bullous pemphigoid	4 (5.00%)	5 (2.46%)	0.472	2.084	0.545–7.969
Cutaneous vasculitis	3 (3.75%)	4 (1.97%)	0.658	1.938	0.424–80861
Cutaneous malignant tumor^†^	1 (1.25%)	4 (1.97%)	1	0.63	0.069–5.722
Linear IgA bullous dermatitis	1 (1.25%)	3 (1.48%)	1	0.844	0.086–8.235
Scleroderma	1 (1.25%)	2 (0.98%)	1	1.272	0.114–14.228
Herpes zoster	0 (0.00%)	4 (1.97%)	0.481	-	-
Erythema multiforme	0 (0.00%)	3 (1.48%)	0.654	-	-
Others^‡^	16 (20.00%)	27 (13.30%)	0.157	1.63	0.824–3.221

CI, confidence interval; OR, odds ratio.

* Pemphigus, including pemphigus vulgaris, pemphigus erythematosus and pemphigus foliaceus.

# Severe drug eruption, including drug-induced exfoliative dermatitis, Stevens-Johnson syndrome, toxic epidermal necrolysis and drug hypersensitivity syndrome.

§ Non-severe drug eruption, including eczematous drug eruption and fixed drug eruption with blisters.

† Cutaneous malignant tumor, including basal cell carcinoma and squamous cell carcinoma.

‡ Others, including stasis dermatitis, necrotizing granuloma, Sweet syndrome, bullous erythema multiforme, lower limb ulcer, Behcet syndrome, furuncle, carbuncle, erysipelas, impetigo, etc.

※ Chi-square test.

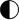
OR from the univariate analysis.

Comorbidities were associated with CA-MRSA, with the top 3 in descending order being pemphigus (26.60%), drug eruption (6.4%), and pustular psoriasis (5.91%). The most common skin disease caused by CA-MRSA was staphylococcal scalded skin syndrome (SSSS) (15.76%). The rate of various comorbidities associated with CA-MRSA did not change significantly between the prior 3 years (2015.1.1–2017.12.31) and the most recent 3 years (2018.1.1–2020.12.31), except that of SSSS, which decreased from 24.24 to 7.45% (*P* = 0.001). The rate of patients with SSSS with CA-MRSA infection was significantly greater than that of patients with HA-MRSA infection (15.76 vs. 2.50%, *P* = 0.002). Regarding comorbidities, the rate of severe drug eruption associated with HA-MRSA was significantly greater than the rate of severe drug eruption associated with CA-MRSA (11.25% vs. 2.46%, *P* = 0.006).

### 3.4. Antibiotic sensitivity profiles

Table 4 lists the antibiotic sensitivities and MIC_50_/MIC_90_ values of the HA-MRSA and CA-MRSA isolates. Overall, both the HA-MRSA and CA-MRSA isolates were sensitive to vancomycin, teicoplanin, tigecycline, linezolid, and daptomycin. The MIC_50_ and MIC_90_ values of these five antibiotics were stable. However, one HA-MRSA isolate exhibited an intermediate phenotype for vancomycin, and one CA-MRSA isolate showed resistance to linezolid. Thus, teicoplanin, tigecycline and daptomycin were the only antibiotics to which all HA-MRSA and CA-MRSA isolates were susceptible. Of the 80 HA-MRSA isolates, 93.75% were resistant to clindamycin, and 96.25% were resistant to erythromycin. Likewise, 80.79% of the CA-MRSA isolates were resistant to clindamycin, and 85.22% were resistant to erythromycin. All the HA-MRSA and CA-MRSA isolates were resistant to penicillin (Table 4). The HA-MRSA isolates were more sensitive to trimethoprim/sulfamethoxazole than the CA-MRSA isolates (88.75 vs. 71.43%; *P* = 0.002; Table 4), and their MIC_90_ values were 8 μg/ml and 16 μg/ml, respectively (Table 4). The CA-MRSA isolates were more sensitive to gentamicin, moxifloxacin, levofloxacin, clindamycin, and erythromycin than the HA-MRSA isolates (*P*-values all < 0.05, and the difference was statistically significant; Table 4).

**Table 4:** Antibiotic susceptibility and MICs of skin and soft tissue healthcare-associated methicillin-resistant *S. aureus* (HA-MRSA) and community-associated methicillin-resistant *S. aureus* (CA-MRSA) isolates from January 1, 2015 to December 31, 2021.

**Antibiotic**	**HA-MRSA**	**CA-MRSA**	*P*-value^*^			**HA-MRSA**		**CA-MRSA**	
	**(*****n** =* **80)**	**(*****n** =* **203)**				**MIC (**μ**g/ml)**		**MIC (**μ**g/ml)**	
	**No. (%)**	**No. (%)**		**OR** [Table-fn TN13]	**95%CI**	**50%**	**90%**	**50%**	**90%**
Vancomycin	79 (98.75)	203 (100)	0.283	-	-	0.5	1	0.5	1
Teicoplanin^#^	33 (100)	77 (100)	1	-	-	0.5	4	0.5	4
Tigecycline	80 (100)	203 (100)	1	-	-	0.125	0.5	0.125	0.5
Linezolid	80 (100)	202 (99.51)	1	-		2	2	2	2
Daptomycin^§^	18 (100)	39 (100)	1	-	-	0.5	1	0.5	1
Rifampicin	59 (73.75)	144 (70.94)	0.636	1.151	0.643–2.062	0.5	2	0.5	4
Trimethoprim/ sulfamethoxazole	71 (88.75)	145 (71.43)	0.002	3.156	1.480–6.730	0.5	8	0.5	16
Gentamicin	46 (57.5)	157 (77.34)	0.001	0.396	0.228–0.688	0.5	16	0.5	16
Moxifloxacin^†^	30 (60.0)	88 (77.88)	0.019	0.426	0.208–0.875	0.25	8	0.25	8
Levofloxacin	45 (56.25)	168 (82.76)	<0.001	0.268	0.151–0.475	0.25	8	0.125	8
Clindamycin	5 (6.25)	39 (19.21)	0.007	0.280	0.106–0.740	8	8	8	8
Erythromycin	3 (3.75)	30 (14.78)	0.009	0.225	0.067–0.759	8	8	8	8
Penicillin	0 (0)	0 (0)	1	-	-	0.5	0.5	0.5	0.5

OR, odds ratio; CI, confidence interval; MIC, minimum inhibitory concentration.

# No sensitivity testing for teicoplanin from 2016 to 2018.

§ No sensitivity testing for teicoplanin from 2015 to 2018.

† No sensitivity testing for moxifloxacin from 2017 to 2018 and some months in other years.

* Chi-square test.

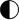
OR from the univariate analysis.

**Figure 2:**
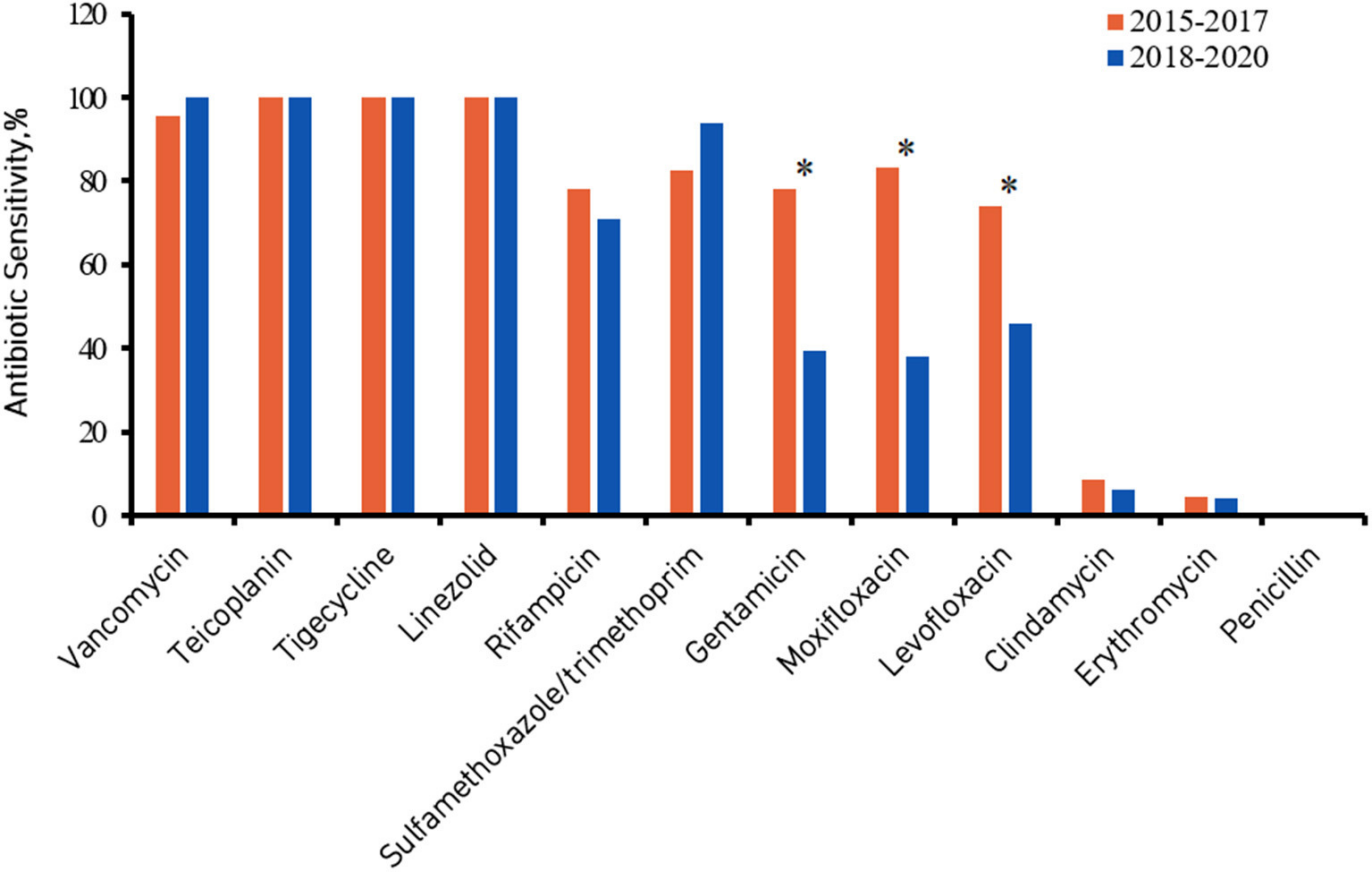
Healthcare-associated methicillin-resistant *Staphylococcus aureus* (HA-MRSA) antibiotic sensitivity from 2015 to 2017 compared with 2018 to 2020. ^*^Denotes a significant difference in antibiotic sensitivity between 2015–2017 and 2018–2020 at *P* < 0.01.

**Figure 3:**
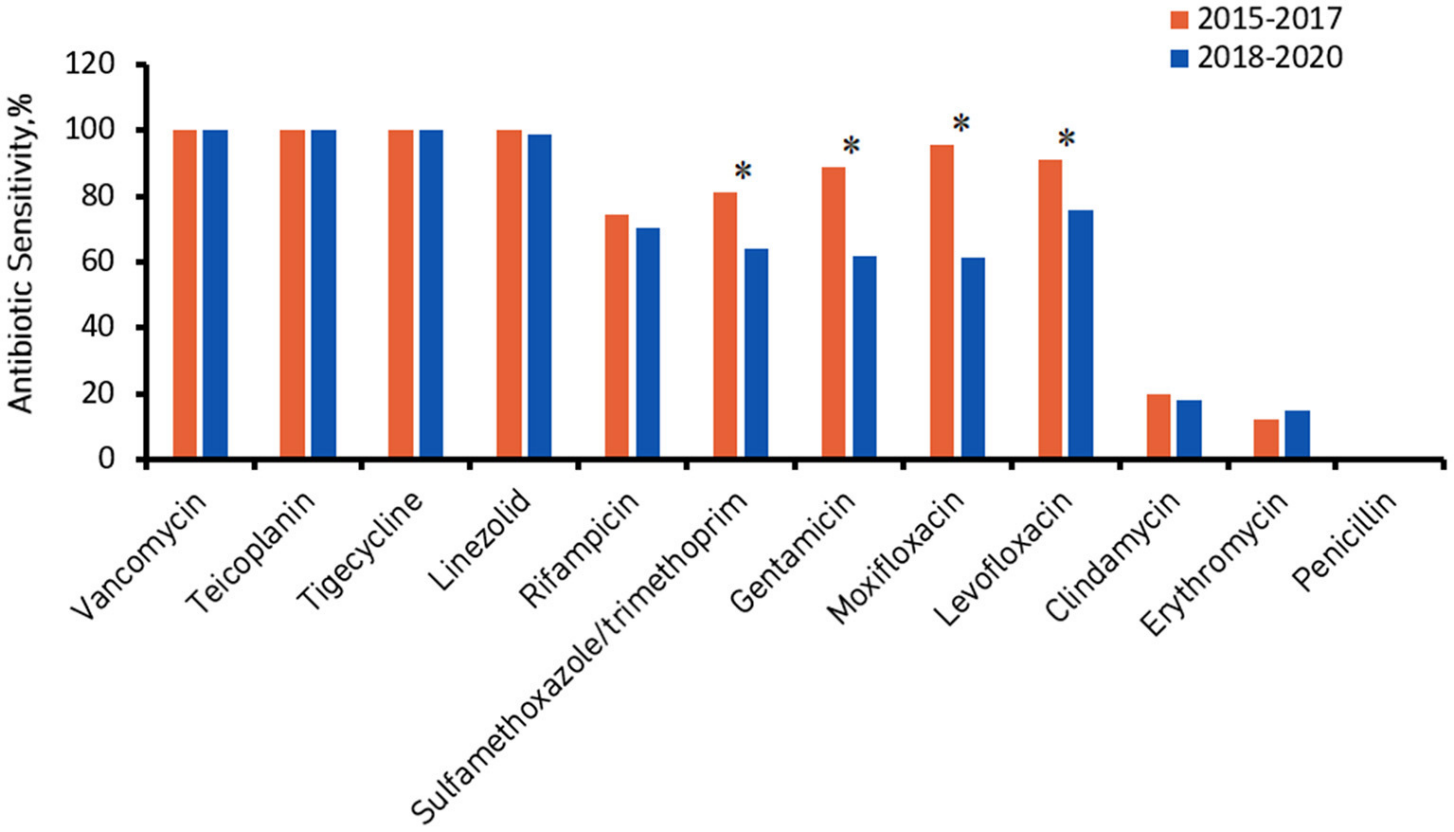
Community-associated methicillin-resistant *Staphylococcus aureus* (CA-MRSA) antibiotic sensitivity from 2015 to 2017 compared with 2018 to 2020. ^*^Denotes a significant difference in antibiotic sensitivity between 2015–2017 and 2018–2020 at *P* < 0.01.

Figures 2, 3 show the comparison of antibiotic sensitivities for the prior 3 years (the period from 2015 to 2017) with the most recent 3 years (the period from 2018 to 2020). According to a comparison of the antibiogram results for these two periods, HA-MRSA in the later period was significantly more resistant to gentamicin, moxifloxacin, and levofloxacin (*P* < 0.01), but the rate of resistance to trimethoprim/sulfamethoxazole decreased (the decrease was not statistically significant) (Figure 2). Comparing the same data for CA-MRSA, the later period showed a significant decrease in antibiotic sensitivity to trimethoprim/sulfamethoxazole, gentamicin, moxifloxacin, and levofloxacin (*P* < 0.01) (Figure 3).

## 4. Discussion

This study was conducted to characterize the prevalence, clinical comorbidities and antibiotic susceptibility of MRSA isolates from SSTIs. To the best of our knowledge, this study represents the largest series of MRSA SSTIs reported in Southwest China. We compared the epidemiology and antibiotic sensitivity of CA-MRSA and HA-MRSA isolates from skin and soft tissue and summarized the comorbidities associated with these two types of SSTI strains, which have not been reported in Southwest China. Our research showed that more than 2/3 of skin and soft tissue MRSA infections were caused by CA-MRSA and that the prevalence of HA-MRSA infection is gradually increasing. All HA-MRSA and CA-MRSA isolates were sensitive to teicoplanin, tigecycline and daptomycin. One CA-MRSA strain was resistant to linezolid, and one HA-MRSA strain had an intermediate phenotype for vancomycin; both strains had low sensitivity to clindamycin and erythromycin. However, HA-MRSA isolates were more susceptible to trimethoprim/sulfamethoxazole. SSSS and severe drug eruption were significantly associated with CA-MRSA and HA-MRSA SSTIs, respectively. These data may help dermatologists prevent MRSA infection in advance and prescribe reasonable antibiotics to treat MRSA (Table 5).

**Table 5:** Clinical outcome associated with skin and soft tissue healthcare-associated methicillin-resistant *S. aureus* (HA-MRSA) and community-associated methicillin-resistant *S. aureus* (CA-MRSA) infection from January 1, 2015 to December 31, 2021.

	**Clinical outcome**										
	**HA-MRSA (*****n** =* **80)**					**CA-MRSA (*****n** =* **203)**					
**Diagnosis**	**No**.	**C**	**I**	**A**	**D**	**No**.	**C**	**I**	**A**	**D**	**Clinical effective rate**[Table-fn TN19] **(%)**
Pemphigus^*^	21	0	19	1	1	54	0	54	0	0	97.33
Staphylococcal scalded skin syndrome	2	2	0	0	0	32	20	12	0	0	100.00
Drug eruption	10	1	9	0	0	13	1	12	0	0	100.00
Severe drug eruption^#^	9	1	8	0	0	5	1	4	0	0	100.00
Nonsevere drug eruption^§^	1	0	1	0	0	8	1	9	0	0	100.00
Pustular psoriasis	5	0	5	0	0	12	0	12	0	0	100.00
Erythroderma	3	0	3	0	0	10	1	9	0	0	100.00
Dermatomyositis	4	0	4	0	0	5	0	5	0	0	100.00
Systemic lupus erythematosus	3	0	3	0	0	5	0	5	0	0	100.00
Eczema	0	0	0	0	0	8	0	8	0	0	100.00
Atopic dermatitis	3	0	3	0	0	6	0	6	0	0	100.00
Pyoderma gangrenosum	3	0	3	0	0	6	0	5	1	0	88.89
Bullous pemphigoid	4	0	4	0	0	5	0	4	0	1	88.89
Cutaneous vasculitis	3	0	3	0	0	4	0	4	0	0	100.00
Cutaneous malignant tumor^†^	1	0	1	0	0	4	1	2	1	0	80.00
Linear IgA bullous dermatitis	1	0	1	0	0	3	0	3	0	0	100.00
Scleroderma	1	0	1	0	0	2	0	2	0	0	100.00
Herpes zoster	0	0	0	0	0	4	0	4	0	0	100.00
Erythema multiforme	0	0	0	0	0	3	0	3	0	0	100.00
Others^‡^	16	0	16	0	0	27	0	27	0	0	100.00

C, cure; I, improvement; A, aggravation; D, death.

* Pemphigus, including pemphigus vulgaris, pemphigus erythematosus and pemphigus foliaceus.

# Severe drug eruption, including drug-induced exfoliative dermatitis, Stevens-Johnson syndrome, toxic epidermal necrolysis and drug hypersensitivity syndrome.

§ Non-severe drug eruption, including eczematous drug eruption and fixed drug eruption with blisters.

† Cutaneous malignant tumor, including basal cell carcinoma and squamous cell carcinoma.

‡ Others, including stasis dermatitis, necrotizing granuloma, Sweet syndrome, bullous erythema multiforme, lower limb ulcer, Behcet syndrome, furuncle, carbuncle, erysipelas, impetigo, etc.

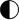
Clinical effectiveness was defined as clinical cure or improvement. Clinical Effective Rate (%) = (C + I)/No. (%).

The prevalence of MRSA varies widely among studies, with reported rates reaching >50% in North America, South America, and Asia (22, 25). A study in Asia showed that the isolation rate for MRSA was 52.5% and that for HA-MRSA was 64% (26). In a national epidemiological study in China, the proportion of MRSA among *S. aureus* infection isolates was 40.16%, with HA-MRSA accounting for 34.63% among MRSA isolates (27). In our study, MRSA accounted for 32.75% of *S. aureus* infections, and HA-MRSA accounted for 28.27% of MRSA infections, which were lower prevalences than those in other Asian and Chinese studies. This discrepancy may be related to the geographic differences in China (28). We found that approximately two-thirds of MRSA SSTIs were caused by CA-MRSA, which is consistent with the predominance of CA-MRSA reported in a recent study in the USA (14, 29). CA-MRSA is also frequently isolated from patients in dermatology departments (30). Most dermatological patients are seen and treated in an outpatient setting, so it is not surprising that CA-MRSA, among MRSA, is important in SSTIs. Importantly, CA-MRSA has gradually become a predominant cause of hospital-acquired infection, which is related to its unique combinations of virulence factors and resistance traits that confer distinct advantages for colonization and pathogenesis (25, 31). CA-MRSA is thus a matter of serious concern, and CA-MRSA infections should be actively prevented. Although the true incidence of CA-MRSA in China is unknown, our data help elucidate the epidemiology of MRSA SSTIs and quantify the extent of CA-MRSA involvement.

In this study, we found that the incidence of HA-MRSA infection increased yearly, reaching 34.78%. We also found that the rate of HA-MRSA among MRSA isolates in the most recent 3 years was significantly higher than that in the prior 3 years. The proportion of HA-MRSA among MRSA isolates is maintained at high levels in some countries in Asia (26). However, there have been no studies on the trend of HA-MRSA SSTIs prevalence in recent years, and our data help clarify the epidemiology of HA-MRSA SSTIs and quantify the extent of HA-MRSA involvement in Southwest China. A study in the USA showed that the incidence of HA-MRSA decreased between 2012 and 2017 (14), and that decrease in incidence was closely related to infection control in health care, such as enhanced contact precautions and effective hand hygiene. This finding also supports strengthening infection control to reduce the incidence of HA-MRSA in our hospital. We also found it interesting that the relative proportion of MRSA among the *S. aureus* isolates in 2020 and 2021 was lower than that in other years, which may be related to the COVID-19 pandemic. It has been reported around the world that the number of cases of MRSA infection in patients with COVID-19 has increased (32, 33). However, in China, we have implemented “Normalized Epidemic Prevention and Control Requirements” (implemented in May 2020), as well as enhanced contact precautions and effective hand hygiene in hospitals, which have reduced the occurrence of MRSA infections (including MRSA SSTI infection) in non-COVID-19 patients (34). Singapore also implemented aggressive infection control strategies during the COVID-19 pandemic, which similarly led to a decline in MRSA infection rates (35). In our study, patients with CA-MRSA infection were younger than those with HA-MRSA infection, which is consistent with other studies (6), with no significant difference in prevalence based on sex.

In terms of the association between MRSA and general comorbidities, previous studies have often addressed various body systems, such as cardiovascular, pulmonary, and neurological systems, and their diseases, while the specific comorbidities of MRSA SSTIs are rarely reported. Therefore, we conducted a subanalysis and discovered that among the comorbidities associated with HA-MRSA and CA-MRSA, pemphigus, drug eruption and pustular psoriasis were the most common. The most common skin disease caused by CA-MRSA infection was SSSS.

Some studies have shown that the existence of open wounds, treatment with antibiotics, use of steroids and immunosuppressant administration are risk factors for HA-MRSA infection (23, 36). The three most common comorbidities (pemphigus, drug eruption (especially severe drug eruption) and pustular psoriasis) share common features, namely, immune disturbances, impaired skin barrier function, and treatment with glucocorticoids and antibiotics in hospitals or in the community, indicating that these three comorbidities confer increased susceptibility to MRSA infection (37–39). Previous studies have suggested that severe drug eruptions lead to increased susceptibility to MRSA infection (38), but our study showed that severe drug eruptions led to a greater increase in susceptibility to HA-MRSA infection than to CA-MRSA infection, which further clarified the type of MRSA infection.

Staphylococcal scalded skin syndrome (SSSS) is a skin disorder characterized by severe blistering and desquamation throughout the body caused by exfoliative toxins (ETs) of *S. aureus*, which occurs most frequently in children. Most epidemiological studies on SSSS showed that MSSA isolates accounted for 98.3–100% of cases in the USA, France, and Ireland (40). However, a recent Korean study showed that MRSA was isolated from 96.2% of patients with SSSS (41). Our study suggests that there are significantly more SSSS patients with CA-MRSA infection than with HA-MRSA infection (15.76 vs. 2.50%), consistent with another study in Taiwan (42). Our study also showed that the incidence of SSSS patients with CA-MRSA infection had significantly decreased in the most recent 3 years compared with that in the prior 3 years. The reason is currently unclear; it may be related to the gradual improvement of diagnosis and treatment in the community. In Southwest China, there is a lack of reports on the specific comorbidities of MRSA SSTIs. Dermatologists may miss the opportunity for early prevention and treatment of MRSA infections because they are unaware of these comorbidities. Therefore, our findings provide a helpful guidance for dermatologists.

Regarding the antibiotic sensitivity analysis, HA-MRSA and CA-MRSA showed different susceptibilities. We observed a notable phenomenon: compared with CA-MRSA, HA-MRSA is more resistant to many antibiotics (such as gentamicin, moxifloxacin, levofloxacin, clindamycin, and erythromycin), which is similar to the results of a cross-sectional study in India (19). A Japanese study showed that gentamicin, moxifloxacin, levofloxacin and clindamycin had high MIC_90_ values against MRSA (128, 64, >256 and >256 μg/ml) (29). However, our results showed relatively low MIC_90_ values (16 μg/ml for gentamicin, and 8 μg/ml for the other antibiotics). HA-MRSA and CA-MRSA isolates were highly sensitive to vancomycin, teicoplanin, tegacyclin, linezolid and datamycin, and the activity of these antibiotics remained stable. According to a comparison of the antibiogram results from 2018 to 2020 with those from 2015 to 2017, HA-MRSA was significantly more resistant to gentamicin, moxifloxacin, and levofloxacin in the later period. However, HA-MRSA showed a significant decrease in resistance to trimethoprim/sulfamethoxazole. Comparing the antibiogram results for the above two periods, it was also found that CA-MRSA was significantly more resistant to trimethoprim/sulfamethoxazole, gentamicin, moxifloxacin, and levofloxacin in the later period.

The increased resistance of HA-MRSA and CA-MRSA to the above antibiotics may be due to the common empirical use of these antibiotics. Empirical antibiotic treatment of HA-MRSA and CA-MRSA infections is often complicated by increased antibiotic resistance, so it is wise to choose antibiotic treatment according to antibiotic sensitivity results. Our study suggests an exception to this trend. We found a significant increase in the sensitivity of HA-MRSA to trimethoprim/sulfamethoxazole, which is different from the results from a study in India (19). A study in Japan showed low MIC_50_ (0.06μg/ml) and MIC_90_ (0.125μg/ml) values for trimethoprim/ulfamethoxazole against MRSA (29). Whereas, in our study, the MIC_50_ and MIC_90_ values were 0.5 μg/ml and 8 μg/ml against HA-MRSA, and 0.5 μg/ml and 16 μg/ml against CA-MRSA. Nevertheless, our data have confirmed that HA-MRSA and CA-MRSA were highly sensitive to this antibiotic, with a sensitivity of 87.65 and 72.14%, respectively. The increased sensitivity to this antibiotic may be related to its reduced use in our hospitals due to its side effects. According to China's National Antibiotic Guide for Skin and Soft Tissue (2015), trimethoprim/sulfamethoxazole can be used empirically to treat MRSA. However, it is best to choose antibiotic therapy based on the results of the latest antibiotic profile. In this study, we conducted treatment according to antibiotic susceptibility, and found that the overall clinical effective rate was 98.59%, and the mortality rate was only 0.71%, which further indicated the importance of treatment according to antibiotic susceptibility.

Although our research has revealed a better antibiotic sensitivity spectrum for HA-MRSA and CA-MRSA, including relatively high sensitivity to common antibiotics and complete sensitivity to teicoplanin, tigecycline and daptomycin, we noted a wide spectrum of antibiotic resistance in both CA-MRSA and HA-MRSA isolates that were resistant to clindamycin and erythromycin. We also found that one HA-MRSA isolate exhibited an intermediate phenotype for vancomycin, and another CA-MRSA isolate showed resistance to linezolid. Nevertheless, vancomycin is still the first option for the treatment of severe MRSA infection. Teicoplanin, tigecycline, daptomycin and linezolid, with similar efficacy, are alternatives due to their toxicity and cost profiles. However, we emphasize that antibiotics should be selected according to the antibiotic profile to reduce the development of antibiotic resistance.

Our study is a retrospective study with some limitations. We distinguished CA-MRSA and HA-MRSA according to epidemiological differences rather than genetic characteristics because our clinical microbiology laboratory did not keep the isolates for a long time. Because our research was conducted in a referral-based tertiary care hospital, the results may not readily reflect the problems in the community. Future research should be multicenter and prospective and use molecular methods to distinguish CA-MRSA and HA-MRSA through genetic characteristics to equally distribute confounders and generalize the results.

## 5. Conclusion

In this 7-year retrospective study, we found that HA-MRSA and CA-MRSA exhibited epidemiological and clinical differences in the Dermatology Inpatient Department of the First Affiliated Hospital of Guangxi Medical University. Our data on the susceptibility of HA-MRSA and CA-MRSA may guide dermatologists in antibiotic treatment decisions to avoid unreasonable use of antibiotics. It is strongly recommended to select appropriate antibiotic treatment plans based on antibiogram results. We summarized the comorbidities most related to CA-MRSA and HA-MRSA SSTIs, which provides an important basis according to disease type for dermatologists to prevent and control MRSA infection in advance. These research data can be applied to clinical dermatology practice and may help to shorten patients' hospital stays and reduce their costs.

## Data availability statement

The raw data supporting the conclusions of this article will be made available by the authors, without undue reservation.

## Ethics statement

This study was approved by the Institutional Review Board at The First Affiliated Hospital of Guangxi Medical University (No. 2022-E430-01).

## Author contributions

CC conceived and designed the study and critically revised the manuscript. YW, HX, YL, WL, ML, YG, and ZJ collected the data. ZY wrote, reviewed, and edited the manuscript. All authors read and approved the manuscript.
